# Lightweight Vision–Transformer Network for Early Insect Pest Identification in Greenhouse Agricultural Environments

**DOI:** 10.3390/insects17010074

**Published:** 2026-01-08

**Authors:** Wenjie Hong, Shaozu Ling, Pinrui Zhu, Zihao Wang, Ruixiang Zhao, Yunpeng Liu, Min Dong

**Affiliations:** 1 China Agricultural University, Beijing 100083, China; 2 National School of Development, Peking University, Beijing 100871, China; 3 School of Economics and Management, Beijing Forestry University, Beijing 100083, China

**Keywords:** pest detection, precision pest management, greenhouse crop protection, lightweight deep learning, intelligent plant health surveillance

## Abstract

This study addresses the challenges of early recognition of fruit and vegetable diseases and pests in facility horticultural greenhouses and the difficulty of real-time deployment on edge devices, and proposes a lightweight cross-scale intelligent recognition network, Light-HortiNet, designed to achieve a balance between high accuracy and high efficiency for automated greenhouse pest and disease detection. The method is built upon a lightweight Mobile-Transformer backbone and integrates a cross-scale lightweight attention mechanism, a small-object enhancement branch, and an alternative block distillation strategy, thereby effectively improving robustness and stability under complex illumination, high-humidity environments, and small-scale target scenarios. Systematic experimental evaluations were conducted on a greenhouse pest and disease dataset covering crops such as tomato, cucumber, strawberry, and pepper. The results demonstrate significant advantages in detection performance, with mAP@50 reaching 0.872, mAP@50:95 reaching 0.561, classification accuracy reaching 0.894, precision reaching 0.886, recall reaching 0.879, and F1-score reaching 0.882, substantially outperforming mainstream lightweight models such as YOLOv8n, YOLOv11n, MobileNetV3, and Tiny-DETR. In terms of small-object recognition capability, the model achieved an mAP-small of 0.536 and a recall-small of 0.589, markedly enhancing detection stability for micro pests such as whiteflies and thrips as well as early-stage disease lesions. In addition, real-time inference performance exceeding 20 FPS was achieved on edge platforms such as Jetson Nano, demonstrating favorable deployment adaptability.

## Simple Summary

This paper presents Light-HortiNet, a lightweight deep learning model tailored for real-time detection of pests and diseases in greenhouse-grown fruits and vegetables. Designed for edge devices like the Jetson Nano, it combines a Mobile-Transformer backbone with cross-scale attention and small-object enhancement techniques to achieve high accuracy under challenging greenhouse conditions—such as variable lighting and high humidity—while maintaining fast inference speeds (over 20 FPS). Experiments show it outperforms existing lightweight models in both overall detection performance and small-target recognition, making it well-suited for practical deployment in smart horticulture systems.

## 1. Introduction

With the continuous advancement of protected horticulture technologies, greenhouse-based fruit and vegetable production has become a key component of high-efficiency global agriculture [1]. Compared with open-field cultivation, greenhouses enable higher yields, extended supply periods, and more stable product quality through precise environmental regulation [2]. Nevertheless, diseases and insect pests remain critical limiting factors for greenhouse productivity. Under typical greenhouse ecological conditions—such as high humidity, low wind flow, and dense planting—pests and pathogens spread rapidly, exhibit strong concealment, and can cause severe damage within short time intervals [3]. Consequently, establishing real-time, accurate, and intelligent recognition systems for greenhouse diseases and pests is of great significance for improving management efficiency, reducing pesticide usage, and ensuring high-quality production.

Traditional disease and pest recognition in greenhouses has mainly relied on manual inspection and expert evaluation [3]. Although such approaches offer a certain degree of reliability, they exhibit several limitations. Manual scouting is labor-intensive and time-consuming, making it difficult to meet the high-frequency monitoring requirements of large-scale modern greenhouses [4]. Moreover, symptoms of diseases and pest infestations in greenhouses often appear similar, atypical in early stages, and concealed by complex plant structures. Variations in individual plant morphology or illumination conditions may lead to misjudgments [5]. The typically complex planting structures of modern greenhouses—such as multilayer leaf occlusions, mixed-cropping systems, reflective films, and supplemental lighting—further reduce the accuracy of manual inspection [6]. Meanwhile, rising labor costs and a decreasing number of agricultural protection specialists have made manual scouting insufficient for intelligent agricultural scenarios that require rapid response and high-precision identification [7].

To address these limitations, researchers have explored classical image-processing techniques, including color threshold segmentation, texture analysis, shape-based descriptors, edge operators, and handcrafted features [8]. Although these methods can identify certain typical symptoms under controlled conditions, their adaptability to complex greenhouse environments is limited [9]. For instance, color-thresholding approaches struggle with illumination-induced color shifts [10]; texture features are highly sensitive to leaf posture variations [11]; and tiny pests such as whiteflies and thrips exhibit small sizes and high morphological similarity, making them difficult to distinguish using handcrafted representations [12]. In addition, rule-based models are highly sensitive to dataset distribution and exhibit weak generalization, often requiring frequent parameter adjustments across crops, greenhouses, or different disease and pest development stages [13]. Thus, traditional image-processing methods are not suitable for long-term, stable deployment in practical greenhouse environments. In recent years, the rapid development of deep learning has significantly advanced intelligent recognition technologies in agriculture. Convolution-based and Transformer-based models have achieved near-expert performance on benchmark datasets such as PlantVillage, and many studies have successfully captured lesion textures, color variations, and pest morphological details using convolutional features and attention mechanisms [14]. Concurrently, object detection frameworks such as YOLO, Faster R-CNN, DETR, and their variants have demonstrated outstanding performance in agricultural disease and pest detection tasks, enabling multi-object recognition in complex greenhouse scenes [15,16]. However, several challenges persist in applying deep learning to greenhouse disease and pest recognition. The greenhouse environment is characterized by strong specular reflections, complex backgrounds, and frequent occlusions, making it difficult for models to capture small-scale and low-contrast details [17]. Furthermore, mainstream deep-learning models typically rely on high-performance computing platforms, posing difficulties for deployment on mobile devices, edge-computing units, greenhouse robots, or smart cameras commonly used in greenhouses [18]. Large networks require high computational resources, making them unsuitable for prolonged operation on low-power devices [19]. Although lightweight models achieve faster inference, their representational capacity is limited and often results in reduced accuracy—particularly in tasks involving small-object detection [20].

Several recent studies have attempted to mitigate these issues. Gao et al. [21] proposed ACLW-YOLO, a lightweight tomato-fruit detection method based on an improved YOLOv11n architecture. The model was compressed to only 3.3 MB while maintaining an mAP of 95.2%, significantly improving deployment efficiency. Zhu et al. [22] developed a fruit disease and pest detection system based on knowledge graphs and deep learning, achieving a pest recognition accuracy of 94.9% on Raspberry Pi devices. Xu et al. [23] introduced CNNA, a lightweight tomato disease and pest classification network based on compressed ConvNeXt-Nano with multi-scale feature fusion and global channel attention, achieving 98.96% accuracy with substantially reduced model size and computational cost. Kong et al. [24] presented LCA-Net, which integrates cross-layer feature aggregation, channel–spatial attention, and Cut-Max cropping, achieving 83.8% accuracy for fine-grained recognition of 28 disease and pest categories. Zhang et al. [25] proposed an automatic greenhouse pest recognition system based on an improved YOLOv5 and machine vision framework, achieving an average accuracy of 96% and significantly enhancing tiny-pest detection capabilities, thereby providing real-time monitoring and decision support for greenhouse pest management.

To address the aforementioned challenges, a lightweight fruit and vegetable disease–pest recognition network named Light-HortiNet is proposed for resource-constrained greenhouse environments. The network is built upon a lightweight Mobile-Transformer architecture and incorporates cross-scale feature interaction and efficient attention mechanisms to maintain high recognition accuracy on low-power platforms.

1. A cross-scale lite attention module (CSLA) is designed, which performs cross-scale information fusion through low-rank decomposition and feature compression, thereby enhancing the model’s ability to capture fine-grained lesion and pest details.
2. A block-level substitution distillation mechanism (BLSD) is introduced, in which intermediate teacher features are used to improve the representational capability of lightweight models without increasing inference cost.
3. A small-object enhancement branch (SOEB) is constructed to strengthen the detection performance on targets of 5–20 pixels while preserving the lightweight structure. This mechanism is particularly effective for tiny pests such as whiteflies and aphids.

## 2. Related Work

### 2.1. Deep Learning Research on Fruit and Vegetable Disease Recognition

In the field of fruit and vegetable disease recognition [26], deep learning has gradually replaced traditional color-, texture-, and shape-based methods and become the mainstream research direction [27]. Convolutional neural networks (CNNs) [28] learn leaf-disease textures, pigment degradation, and edge morphology through stacked convolution and pooling operations [29], achieving near-expert classification accuracy on benchmark datasets such as PlantVillage [30]. As agricultural in-field datasets expanded, models were progressively transferred from controlled laboratory environments to real greenhouse and field scenarios [31]. Accordingly, Vision Transformer (ViT), Swin Transformer, and related architectures have been applied to disease classification and lesion localization tasks [32]. However, these models typically require large computational budgets and a high number of parameters [33]. Their dependence on powerful GPUs or cloud-based environments limits their suitability for real-time deployment on greenhouse edge devices. Even when achieving high accuracy, inference latency tends to be prohibitive for practical use [34]. Many features often lie at fine-grained spatial scales, making them difficult for standard CNN or Transformer architectures to capture without specialized design [35]. Thus, although deep learning-based disease recognition methods perform well in high-compute scenarios, they remain constrained in resource-limited greenhouse environments dominated by small-scale and detail-sensitive targets, highlighting the need for lightweight cross-scale feature modeling strategies as proposed in this work.

### 2.2. Agricultural Pest Detection and Tiny-Object Recognition

To address tiny-object detection [36,37], researchers have adopted TinyObject Detection techniques, including enhanced feature pyramid structures, higher-resolution feature maps, and additional high-resolution branches to improve recognition performance [38]. YOLO models, due to their real-time performance, are widely used in agricultural detection tasks. The YOLOv5, YOLOv8, and their nano variants maintain high detection speed under lightweight configurations. However, their backbone–neck designs are fundamentally optimized for medium-scale objects, leading to frequent missed detections for micro-scale pest bodies [39]. Furthermore, when pests blend into leaf textures or appear in dense clusters, the single-scale anchor mechanism of YOLO architectures cannot sufficiently model such complex distributions [40]. On the other hand, DETR and its variants employ Transformer decoders to achieve end-to-end detection with strong robustness to complex backgrounds. Nevertheless, DETR suffers from long inference latency and slow convergence and still performs suboptimally on tiny-object detection compared with feature pyramid-based detectors, making it unsuitable for direct deployment on greenhouse mobile platforms or smart cameras [41].

### 2.3. Lightweight Models and Edge Deployment

Research in lightweight architectures and edge deployment has formed a relatively mature technical foundation [42,43]. Architectures such as MobileNet and ShuffleNet reduce computational cost through depthwise separable convolution, pointwise convolution, and group convolution, enabling real-time inference on mobile devices [44]. However, in greenhouse disease–pest recognition tasks, purely lightweight convolutional operations [45] are generally insufficient to capture the fine-grained patterns of complex lesions and tiny pests. Their recognition capability remains substantially lower than that of large-scale models [46]. Additionally, knowledge distillation has emerged as an effective approach to improving lightweight model performance without increasing inference cost. By allowing a lightweight student model to learn intermediate features or output distributions from a heavier teacher model, representational capacity can be significantly enhanced [47]. Significant progress has been made in disease recognition, pest detection, tiny-object processing, and lightweight deployment [48]. Nevertheless, in resource-constrained greenhouse environments where sensitivity to small targets is essential, several limitations persist.

## 3. Materials and Method

### 3.1. Data Collection

**Figure 1:**
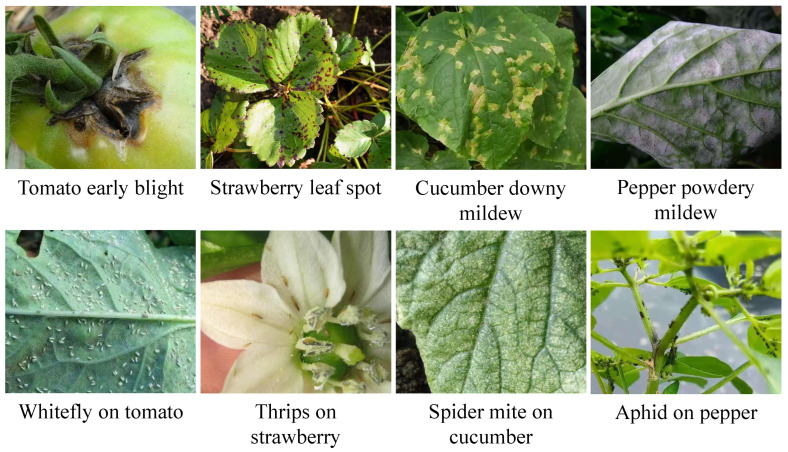
Illustration of the greenhouse crop disease and insect pest data collection.

**Figure 2:**
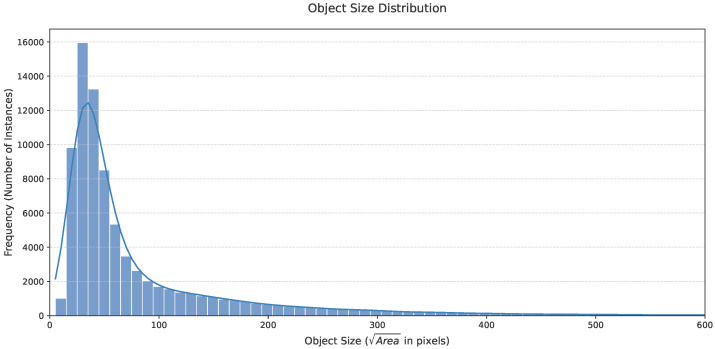
Size distribution histogram.

The dataset constructed in this study was derived from three complementary channels, including in situ greenhouse acquisition, supplementation from open-access online sources, and expert-verified annotation integration, aiming to comprehensively cover the typical visual characteristics of diseases and pests in fruit and vegetable greenhouse environments. As shown in Table 1 and Figure 1, the field data were mainly collected from March 2022 to October 2024, covering representative solar and multi-span greenhouse environments in North China, East China, and Southwest China, and the target crops included tomato, cucumber, strawberry, and pepper, which are common facility horticultural species. Image acquisition was conducted using high-resolution rgb industrial cameras and mobile terminal devices, with image resolutions ranging from 1920×1080 to 4032×3024 pixels. The collection process strictly simulated natural and supplementary lighting conditions in real greenhouse production environments, including low-angle incident light in the morning, high-intensity direct illumination at noon, and diffuse weak-light scenes in the evening, while deliberately preserving real visual noise characteristics such as water vapor interference caused by high humidity, leaf specular reflection, and complex soil background patterns. The shooting strategy combined multi-angle close-range circular imaging with random viewpoint acquisition, and pest samples were mainly captured with close-up views focusing on the abaxial leaf surfaces, stem junction regions, and floral organ surroundings, in order to preserve the authentic scale distribution characteristics of micro-scale pests. Distribution at the scale of micro pests is shown in Figure 2.

The online dataset were primarily collected from publicly available agricultural disease and pest databases and open-source visual datasets, including international plant disease repositories and internet-based agricultural pest image sharing platforms, with a collection period spanning from 2020 to 2025. This component of the dataset was mainly used to complement rare pest categories and early-stage lesion samples, and the corresponding images were characterized by diverse background environments and significant differences in imaging devices, exhibiting pronounced cross-domain distribution properties, which contributed to improving the generalization robustness of the model. All image samples were double-checked and annotated by plant protection experts with extensive field experience, and boundary box drawing and category confirmation were assisted by semi-automatic annotation tools. A dual-annotator cross-validation strategy was adopted during the labeling process to reduce human-induced errors, and the annotation format was unified to a standard object detection format. In addition, metadata including crop variety, growth stage, and acquisition environmental conditions were recorded to ensure dataset traceability and scientific rigor.

### 3.2. Data Augmentation

In greenhouse disease and pest recognition tasks, data quality was found to directly influence model robustness and generalization performance. Greenhouse environments are characterized by strong illumination fluctuations, pronounced local reflective highlights, severe leaf overlap, complex background textures, and a high proportion of small targets. Therefore, targeted data augmentation strategies were required to enable effective learning of the feature distributions of disease lesions and micro-scale pests under high-noise and high-variability imaging conditions. In this study, factors including color variation, spatial interference, scale variation, and small-target sparsity were comprehensively considered during data preprocessing, and a series of augmentation techniques were adopted to construct high-quality training data suitable for greenhouse scenarios.

#### 3.2.1. Basic Augmentation

First, color jittering was applied to simulate complex and rapidly changing illumination conditions in greenhouses. Due to supplementary lighting, plastic film reflections, and shading net occlusion, leaf colors in different regions can present significant differences in brightness and saturation. To enhance robustness against illumination drift, random perturbations of brightness, contrast, saturation, and hue were applied to the images. Let the original image be denoted as *I*, and the color-transformed image can be expressed as

(1) $$I^{\prime }=T_{\mathrm{color}}\left(I;\alpha ,\beta ,\gamma ,\delta \right),$$

where α, β, γ, and δ correspond to random coefficients of brightness, contrast, saturation, and hue, respectively. Since lesion color variations are usually subtle, such as the slight grayish appearance in the early stage of downy mildew, color jittering encourages the model to focus on more stable texture and structural information rather than incidental background illumination features. Subsequently, to simulate common leaf occlusion and organ overlap in greenhouses, random occlusion and cutout operations were introduced. The core idea was to generate an occlusion region of fixed or variable size at random locations in the image, thereby forcing the model to learn contextual information rather than relying on a single local feature. Let the occlusion region be denoted as *M*, and its distribution over the image can be expressed as

(2) $$I^{\prime }=I⊙\left(1-M\right),$$

where ⊙ denotes pixel-wise multiplication. The occlusion mask *M* is typically a binary matrix, and pixel values within the covered regions are set to constant or noise values. Greenhouse pests such as aphids and thrips are often partially occluded due to complex leaf structures; thus, cutout operations enhance model generalization to incomplete target features. Similarly, lesions near leaf veins are frequently partially occluded, and random occlusion encourages the network to learn more robust disease patterns.

#### 3.2.2. Small-Object Enhancement

**Figure 3:**
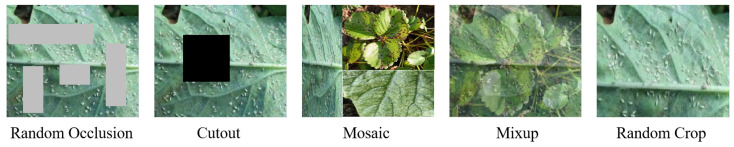
Illustration of small-object enhancement strategies.

To enhance learning capability for small targets such as pests, random crop and resize strategies were further adopted, as shown in Figure 3. Specifically, pest sizes are typically within the range of 5–20 pixels relative to the standard model input resolution (e.g., 640×640). When full high-resolution images are directly used as input, pests may occupy only a very small region, making effective feature extraction difficult.

By randomly cropping regions containing pests and resizing them to the model input size, the effective pixel proportion of small targets can be significantly increased. Let the original image region be denoted as *I* and the cropping window as *W*, then the cropping process can be expressed as

(3) $$I^{\prime }=\mathrm{Resize}\left(\mathrm{Crop}\left(I,W\right),H\times W\right),$$

where H×W denotes the model input resolution. This operation not only increases the prominence of pest features but also ensures cross-scale learning capability, which is critical for improving small-object detection accuracy. Similarly, the fusion of pests of different scales in dense pest regions further improves the learning of different pest growth stages. In addition, mosaic and mixup, which have demonstrated significant effectiveness in recent object detection tasks, were adopted to enrich scene combinations and enhance spatial understanding ability. Mosaic stitches four images together according to random proportions, thereby improving adaptability to different backgrounds and disease–pest combinations. Let the four images be denoted as $I_{1},I_{2},I_{3},I_{4}$, and the mosaic composition can be expressed as

(4) $$I^{\prime }=\mathrm{Concat}\left(I_{1},I_{2},I_{3},I_{4}\right).$$

Mixup linearly mixes two images to smooth the data distribution and improve generalization capability, and can be expressed as

(5) $$I^{\prime }=\lambda I_{a}+\left(1-\lambda \right)I_{b},$$

where λ∼Beta(α,α) denotes the mixing coefficient. In greenhouse scenarios, mixup effectively reduces overfitting caused by illumination reflections and leaf texture variations, enabling the network to learn more general spatial and color features.

Finally, to address the critical issue that pests and early-stage lesions commonly exhibit small-scale characteristics, a small-object copy–paste strategy was introduced. Since micro-scale pests usually occupy an extremely low proportion of the original image, direct training tends to bias the model toward background pattern learning while neglecting critical targets. To alleviate class imbalance and small target scale problems, small objects were obtained through detection or annotation, then cropped, copied, and pasted into images of the same or different categories, thereby enriching spatial distribution and scale variation. Let the small object region be denoted as *o* and the target image as *I*, and the enhancement can be expressed as

(6) $$I^{\prime }=I+\sum_{i=1}^{N}S_{\lambda_{i}}\left(o_{i}\right),$$

where $S_{\lambda_{i}}$ denotes the scaling operation with scale factor $\lambda_{i}$, and *N* denotes the number of pasted small objects. Through appropriate scale perturbation and position randomization, the perception capability of small lesions and pests can be greatly improved, enabling more accurate recognition of micro-scale targets in real greenhouse environments during inference.

### 3.3. Proposed Method

#### 3.3.1. Cross-Scale Lightweight Attention Module

**Figure 4:**
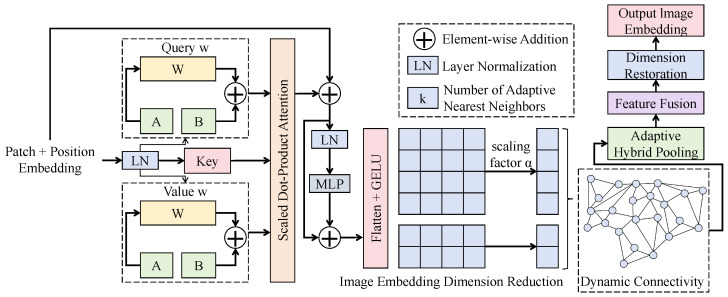
Structure of the cross-scale lightweight attention (CSLA) module.

The cross-scale lightweight attention module (CSLA) exhibits fundamental differences from conventional self-attention mechanisms in terms of design objectives and computational pathways. Standard self-attention typically constructs global correlation modeling within a single-scale feature space, and the attention matrix scale increases quadratically with input resolution, which makes it difficult to adapt to resource-constrained devices. In contrast, CSLA introduces cross-scale feature compression and low-rank approximation mechanisms to construct sparse correlation modeling paths across multi-scale feature spaces, thereby transforming attention computation from global dense mapping to local cross-layer hybrid mapping. As shown in Figure 4, for an input feature map $X\in R^{H\times W\times C}$, CSLA first constructs compact query, key, and value matrices through three groups of lightweight mapping layers, which are defined as

(7) $$Q=XW_{Q},\;K=XW_{K},\;V=XW_{V},$$

where $W_{Q},W_{K},W_{V}\in R^{C\times C^{\prime }}$ and $C^{\prime }=C/4$. Subsequently, a scale compression operator S(·) is introduced to downsample the feature maps into multi-scale spaces, and cross-scale attention weights are constructed as

(8) $$A_{cs}=\mathrm{Softmax}\left(\frac{Q_{s}K_{s}^{T}}{\sqrt{d_{s}}}\right),$$

Unlike the single-scale $QK^{T}$ computation in standard self-attention, CSLA constructs multi-scale subspaces with s∈{1,2,4}, thereby significantly reducing computational complexity and enhancing the perception capability for small-object structures.

In terms of structural design, CSLA adopts a three-level nested architecture embedded within each Mobile-Transformer block of the backbone. The input dimensions of the first level are fixed as H/4×W/4×64, the second level as H/8×W/8×96, and the third level as H/16×W/16×128. Each level internally employs depthwise 3×3 convolution and pointwise 1×1 convolution to construct lightweight mapping units, while low-rank decomposition constraints are introduced to approximate the attention matrix as the product of two rank-constrained matrices:

(9) $$A_{cs}\approx UV^{T},\;U\in R^{n\times r},\;V\in R^{m\times r},\;r\ll \min\left(n,m\right).$$

Through this construction, the complexity is reduced from $O({\left(HW\right)}^{2})$ in conventional self-attention to O(HW·r). The proof is based on matrix rank constraint theory, and when *r* is fixed and much smaller than HW, the upper bound of the approximation error for the principal feature subspace satisfies

(10) $$|A-UV^{T}{|}_{F}\leq \sum_{i=r+1}^{\min(n,m)}\sigma_{i},$$

where $\sigma_{i}$ denotes the singular values of the original attention matrix, thereby achieving significant compression while preserving the main semantic information.

In the overall network, CSLA is collaboratively integrated with the lightweight Mobile-Transformer backbone in a parallel embedded fusion manner. Specifically, the backbone convolutional features are first processed by local convolutional encoding and then fed into the CSLA module for cross-scale saliency recalibration, followed by residual connections back to the backbone feature channels, so that semantic enhancement is achieved without altering the original feature topology. The theoretical basis of this joint design is that, under the residual learning framework, the feature perturbation term satisfies the stability constraint:

(11) $$Y=X+f_{\mathrm{CSLA}}\left(X\right),\;\frac{\partial Y}{\partial X}=I+\frac{\partial f}{\partial X},$$

When $\left|\frac{\partial f}{\partial X}\right|\leq 1$, gradient propagation in the network remains stable, thereby ensuring that the lightweight model does not suffer from gradient vanishing or explosion as training depth increases. Through this approach, CSLA can be deeply coupled with the backbone network under extremely low additional overhead, while significantly enhancing cross-scale modeling capability for lesion edges and pest regions, thereby providing high-quality foundational attention feature representations for subsequent BLSD and SOEB modules.

#### 3.3.2. Alternative Block Distillation Module

The core concept of the alternative block distillation module (BLSD) is to introduce block-level semantic substitution and mapping constraints into the hierarchical structure of a lightweight backbone network, so that the student model can absorb the layer-wise semantic representation capability of a high-capacity teacher model at extremely low computational cost during training. Regarding the teacher model selection, we employed a high-capacity variant of the Mobile-Transformer architecture to ensure structural isomorphism with the student network, thereby facilitating precise block-to-block feature alignment. This teacher network was initialized with ImageNet-1K pre-trained weights and fine-tuned on the target greenhouse dataset to guarantee robust feature extraction and convergence stability before distillation. Specifically, the teacher model contains N=12 transformer blocks with channel dimensions of {64,96,128,192}, and the corresponding spatial resolutions are H/4×W/4, H/8×W/8, H/16×W/16, and H/32×W/32. The student model adopts the same four-stage structure as the Mobile-Transformer backbone, while the channel numbers of each stage are reduced to 1/2 of the teacher model, i.e., {32,48,64,96}. During the distillation process, both networks maintain identical spatial resolutions, so that only linear adjustment along the channel dimension is required during the substitution mapping.

**Figure 5:**
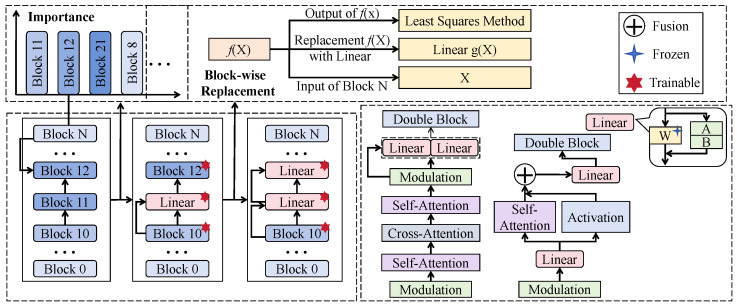
Illustration of the alternative block distillation (BLSD) module.

As shown in Figure 5, for the output feature of the *i*-th block of the teacher model $T_{i}\in R^{H_{i}W_{i}\times C_{i}}$ and the corresponding block feature of the student model $S_{i}\in R^{H_{i}W_{i}\times C_{i}^{\prime }}$, BLSD establishes semantic alignment through a trainable linear mapping matrix $M_{i}\in R^{C_{i}^{\prime }\times C_{i}}$, so that the substitutability of student features can approximate teacher features. The mathematical form is expressed as

(12) $${\hat{T}}_{i}=S_{i}M_{i},$$

and a block-level distillation constraint is constructed by minimizing the substitution error as

(13) $$L_{\mathrm{blk}}^{\left(i\right)}={\left|T_{i}-{\hat{T}}_{i}\right|}_{2}^{2}.$$

In the alternative block mechanism, teacher features are not only involved in error backpropagation as constraint terms, but can also directly replace the outputs of certain blocks of the student model during the early training stage, forming a substitution path $R_{i}$. For the *k*-th block of the student model, the input can be dynamically selected from the output of the previous student layer $S_{k-1}$ or the mapped teacher output $T_{k-1}$, and is generated through the gating function

(14) $$Z_{k}=\alpha_{k}T_{k-1}+\left(1-\alpha_{k}\right)S_{k-1},$$

where $\alpha_{k}\in \left[0,1\right]$ is an adaptive weight jointly determined by importance ranking and gradient stability. To demonstrate that the substitution path does not destroy model stability, the block energy variation can be defined to satisfy

(15) $$|Z_{k}-S_{k-1}{|}_{2}\leq \alpha_{k}{\left|T_{k-1}-S_{k-1}\right|}_{2},$$

and, since the teacher model has stronger representation capability, the upper bound of the right-hand error term can be guaranteed to converge, so that the substitution path adaptively tends to $\alpha_{k}\to 0$ in the later training stage, thereby proving the asymptotic vanishing property and training stability of the substitution mechanism. The internal substitution linear structure of BLSD adopts a bilinear layer stacking as shown in the figure, with a channel expansion ratio of r=4, and its parameter scale is $C_{i}^{\prime }\times \left(rC_{i}^{\prime }\right)+\left(rC_{i}^{\prime }\right)\times C_{i}$, which is significantly lower than the multi-head attention structure of a standard transformer block. Each substitution block includes substructures such as modulation, self-attention, cross-attention, and activation, while only the linear substitution path and modulation module are retained in the student network and the remaining parts are frozen to reduce computation. The depth of the substitution block is fixed as two linear layers plus one modulation layer, and the overall FLOPs (floating point operations per second) increment accounts for only 3.2% of the backbone network.

#### 3.3.3. Small-Object Enhancement Branch

**Figure 6:**
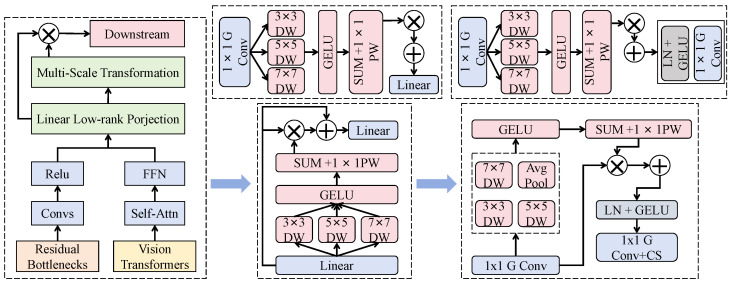
Illustration of the small-object enhancement branch (SOEB).

The small-object enhancement branch (SOEB) is designed to preserve salient information of pest bodies at 5–20 pixel scale and early-stage lesion regions in greenhouse environments, and a high-resolution parallel subnetwork is adopted to avoid detail loss caused by backbone downsampling. As shown in Figure 6, this branch diverges from shallow backbone feature maps, with an input size of H/4×W/4×64, and is first compressed to 48 channels through a 1×1 group convolution, followed by a three-layer stack of depthwise separable convolutions with kernel sizes of 3×3, 5×5, and 7×7. All strides are set to 1, and spatial resolution is preserved, while channel dimensions are progressively expanded to 64, 80, and 96, respectively. Subsequently, GELU activation and pointwise convolutions are introduced for channel reorganization, so that the output features are stably maintained at a tensor scale of H/4×W/4×128. At the intermediate stage, a linear low-rank projection is employed to map high-dimensional features into a compact space, thereby reducing the computational burden during subsequent fusion. The mapping process can be expressed as

(16) $$Z=XW_{1}W_{2},\;W_{1}\in R^{C\times r},\;W_{2}\in R^{r\times C^{\prime \prime }},$$

where *r* is set to C/8 to ensure that major structural information is preserved under the rank constraint.

With respect to the feature enhancement mechanism, SOEB adopts joint modeling of multi-scale transformations and adaptive pooling. The features after depthwise convolution stacking are processed by 3×3 and 5×5 depthwise convolution branches, and are complemented by a 7×7 depthwise convolution and average pooling to form a local–global dual-path structure. Finally, element-wise summation and a 1×1 pointwise convolution are applied to complete feature recalibration. This process can be formalized as

(17) $$E=\phi \left(\sum_{k\in \{3,5,7\}}D_{k}\left(Z\right)\right)\cdot \alpha +P\left(Z\right)\cdot \left(1-\alpha \right),$$

where $D_{k}\left(\cdot \right)$ denotes a depthwise convolution operator with kernel size *k*, P(·) denotes the average pooling operator, and α is a learnable gating parameter. According to the theory of multi-scale response superposition, it can be demonstrated that this fusion form satisfies the feature amplification property, that is, when the target size approaches the minimum scale, the expected value of the convolutional response satisfies

(18) $$E\left[E_{s}\right]\geq E\left[Z_{s}\right],$$

thereby ensuring that the activation probability of tiny targets in the feature space does not decay as the scale decreases.

In the overall network, SOEB forms a cascaded collaborative structure with CSLA and BLSD. The cross-scale attention weights generated by CSLA are injected into the modulation layers of SOEB as guiding signals, so that the high-resolution branch focuses on high-confidence regions in the spatial dimension. Meanwhile, the semantic substitute features provided by BLSD during training are injected into the channel modulation module of SOEB through linear mapping, enabling shallow features to be constrained by high-level semantics. The joint mapping relationship can be expressed as

(19) $$U=E+\eta \cdot G_{cs}+\mu \cdot D_{blk},$$

where $G_{cs}$ denotes the output features of CSLA, $D_{blk}$ denotes the distilled features generated by BLSD, and η and μ are learnable fusion weights. Through this design, the recall stability for small pest bodies and early-stage lesions is significantly improved in greenhouse pest and disease tasks, and high-sensitivity amplification of tiny targets is achieved without a significant increase in model parameters and FLOPs, thereby enabling the lightweight model to maintain reliable small-object perception capability on edge devices.

## 4. Results and Discussion

### 4.1. Experimental Configuration

#### 4.1.1. Hardware and Software Platform

The experimental hardware platform in this study was designed to cover a wide spectrum ranging from high-performance training servers to resource-constrained edge computing devices, ensuring deployability and stability across diverse application scenarios. During the high-performance training stage, NVIDIA A100 and RTX 4090 GPUs were utilized, providing powerful tensor-core computation capabilities and high-bandwidth memory resources, which enabled large-batch training and parallel multi-model experimentation. For edge inference evaluation, Jetson Nano and RK3588 were selected as representative low-power embedded platforms to validate real-time response capability in practical farmland and greenhouse environments, while a mobile-side NPU was further employed to assess inference latency and energy consumption on mobile devices. Through the cross-platform experimental design, systematic evaluation of performance consistency and deployment adaptability under heterogeneous computational conditions was achieved. The software environment was established based on the PyTorch 1.10.0 deep learning framework for model training and optimization, where the dynamic computation graph facilitated rapid prototyping and customized module development. During the inference acceleration stage, TensorRT was adopted for operator optimization and quantization to further reduce inference latency on edge devices, while ONNX Runtime was employed to enable cross-platform model inference, maintaining structural consistency between training and deployment pipelines and thereby reducing compatibility issues during model migration. The overall software stack covered training, exporting, optimization, and deployment stages, ensuring controllability and portability across the full model lifecycle.

#### 4.1.2. Hyperparameter Settings

With respect to hyperparameter configuration, the dataset was divided into training, validation, and test sets with a ratio of 7:2:1. To ensure representational fairness across all categories, a stratified sampling strategy was employed during the partitioning process. This mechanism guaranteed that the distribution of disease and pest classes in each subset remained consistent with the overall dataset, effectively mitigating evaluation bias caused by potential class imbalance and ensuring that minority classes were sufficiently represented in the validation and test phases. During training, a batch size of batch_size = 32 was adopted, the initial learning rate was set to $\alpha =1\times 10^{-3}$, and a cosine annealing strategy was applied for dynamic learning rate adjustment, while the AdamW optimizer was employed to achieve more stable gradient update behavior. To enhance model generalization capability, five-fold cross-validation was introduced, where the dataset was partitioned into five subsets and one subset was selected as the validation set in each iteration while the remaining four subsets were used for training, and a total of f=5 cycles were conducted to obtain more robust and unbiased performance estimation. The above combination of hyperparameters enabled stable convergence behavior and strong generalization performance under varying conditions, providing a reliable foundation for subsequent comparative experiments and deployment verification.

#### 4.1.3. Baseline Models and Evaluation Metrics

The baseline models selected in this study covered representative lightweight detection and visual backbone networks commonly adopted in agricultural small-object scenarios, including YOLOv8n [49], YOLOv11n [50], YOLOX-Tiny [51], MobileViT [52], MobileNetV3 [53], EfficientDet-D0 [54], and Tiny-DETR [55]. To ensure the fairness and validity of the performance comparison, all baseline models were trained and evaluated under identical experimental conditions, strictly adhering to the same dataset partitioning strategies, input resolutions, and optimization hyperparameter settings.

Model performance evaluation was conducted using mAP@50 and ${\mathrm{mAP}}_{50:95}$ for detection tasks, accuracy and f1-score for classification tasks, ${\mathrm{mAP}}_{small}$ for small-object detection performance, inference speed metrics including fps and model size (mb) as well as FLOPs, and real deployment latency and power consumption to assess comprehensive performance on edge devices. In mathematical definitions, the core evaluation formulas for detection and classification were uniformly expressed as follows:

(20) $$precision=\frac{TP}{TP+FP},$$

(21) $$recall=\frac{TP}{TP+FN},$$

(22) $$f1=2\times \frac{precision\cdot recall}{precision+recall},$$

(23) $$accuracy=\frac{TP+TN}{TP+TN+FP+FN},$$

(24) $$AP=\int_{0}^{1}P\left(R\right),dR,$$

(25) $$mAP=\frac{1}{N}\sum_{i=1}^{N}AP_{i},$$

(26) $$FPS=\frac{1}{t_{infer}},$$

(27) $$FLOPs=\sum_{l=1}^{L}C_{l}\cdot H_{l}\cdot W_{l}\cdot K_{l}^{2}.$$

The variables in the above formulas were defined as follows: TP, FP, TN, and FN denote the numbers of true positives, false positives, true negatives, and false negatives, respectively; P(R) represents the precision curve at different recall rates *R*; *N* denotes the number of categories; $t_{infer}$ denotes the inference time per image; *L* denotes the number of network layers; $C_{l}$, $H_{l}$, and $W_{l}$ denote the channel number and feature map dimensions of the *l*-th layer; and $K_{l}$ denotes the convolution kernel size. In a comprehensive sense, detection metrics mainly measured the combined performance of localization and classification, classification metrics reflected the accuracy of disease symptom recognition, small-object metrics emphasized the detection capability of fine-grained pest targets, speed-related metrics reflected real-time capability under resource-constrained environments, and deployment performance further demonstrated practical value in real agricultural edge scenarios, ensuring that the model was required not only to be accurate, but also to be fast and efficient.

### 4.2. Overall Performance Comparison

The objective of this experiment was to systematically evaluate the comprehensive perception capability of different lightweight visual models under complex greenhouse fruit and vegetable disease and pest scenarios. Through unified data partitioning and inference settings, the balance between detection accuracy, classification performance, and inference efficiency was compared, thereby validating the practicality and stability of the proposed model in resource-constrained environments.

**Figure 7:**
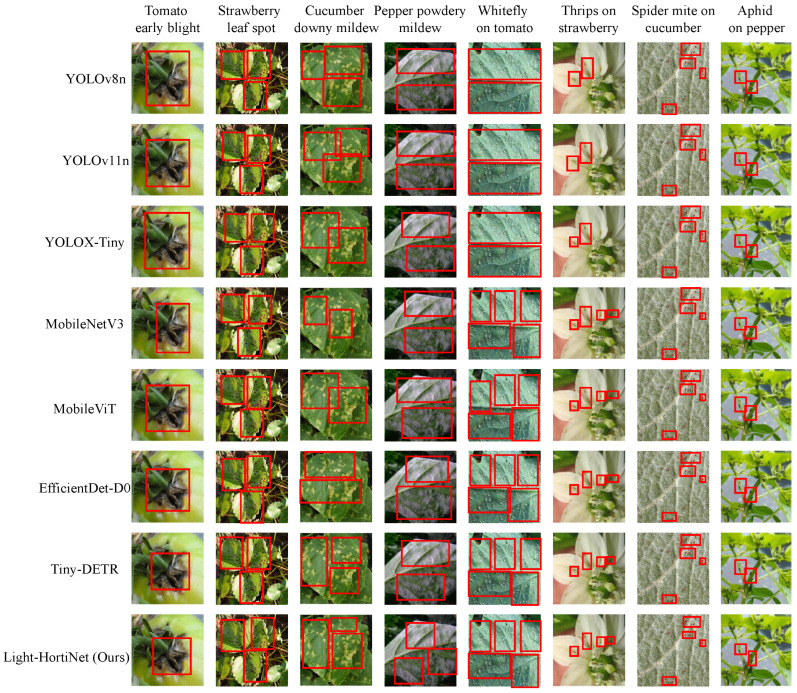
Visualization of test results.

From the overall results, as shown in Table 2 and Figure 7, the highest values of mAP@50, mAP@50:95, accuracy, and f1-score were achieved by light-hortinet, while a relatively high inference frame rate was maintained under a controlled parameter scale, indicating that a more favorable balance between accuracy and efficiency was realized. YOLOv8n and YOLOv11n, as representative one-stage detection frameworks, exhibited stable accuracy due to strong local feature modeling capability; however, fixed-scale feature fusion mechanisms still resulted in information loss when facing small targets and complex backgrounds in greenhouse environments. YOLOX-tiny and mobilenetv3 adopted extremely lightweight designs, and computational complexity was reduced through channel pruning and depthwise separable convolutions, resulting in higher fps performance, but feature representation dimensionality was constrained, leading to a pronounced decline in accuracy for fine-grained lesion and pest recognition. Mobilevit enhanced global modeling capacity by introducing a lightweight transformer structure, yet scale coupling issues remained in attention computation, which limited performance in high-density small-object scenarios. Efficientdet-d0 relied on bidirectional feature pyramid networks to enhance multi-scale fusion, but under extreme lightweight configurations, channel capacity was restricted, leading to insufficient feature reuse efficiency. Tiny-detr was designed based on a set-matching detection paradigm, but its global attention structure was unable to sufficiently capture local high-frequency textures under low-resolution embedding conditions, resulting in weaker performance in both accuracy and speed.

**Figure 8:**
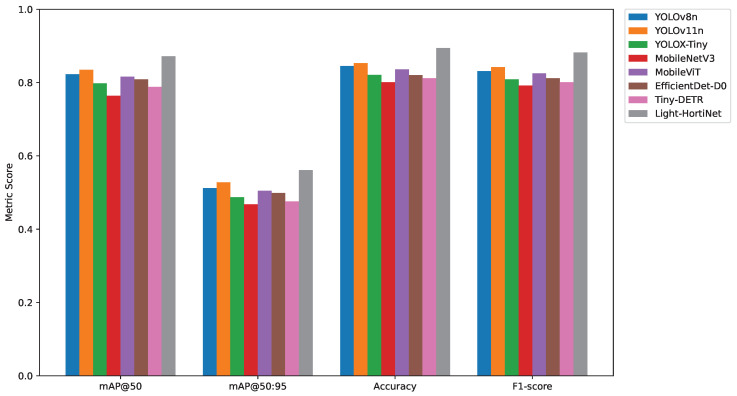
Performance comparison with lightweight baseline models.

As shown in Figure 8, the significant advantage of light-hortinet was attributed to the introduction of multi-level low-rank constraints and cross-scale sparse modeling mechanisms in the feature mapping space, which caused the feature distributions to become more concentrated and the decision boundaries to become clearer. Traditional YOLO series models relied on convolutional kernels with fixed receptive fields, and their feature responses were mainly formed by the superposition of local linear transformations, which ensured translational invariance but led to feature aliasing under complex illumination and occlusion conditions. Mobilenet series models reduced parameter scale by depthwise separable convolution, which can be interpreted as an implicit rank-constrained approximation of convolution operators, improving speed but weakening the expressive freedom of the high-dimensional feature space. Transformer-based models relied on global correlation matrix modeling to capture long-range dependencies, but under lightweight constraints, the compression of attention matrix dimensions resulted in reduced discriminative capability. In contrast, light-hortinet formed a more stable low-dimensional manifold structure in the feature space through cross-scale lightweight attention and block-level distillation mechanisms, while the small-object enhancement branch strengthened local high-frequency response. Consequently, effective compression of feature information entropy and maximal preservation of discriminative information were achieved at the mathematical level, which constituted the fundamental reason why superior accuracy and efficiency were simultaneously obtained under a compact model scale.

### 4.3. Comparison of Small-Object Detection Capability

The objective of this experiment was focused on evaluating the perception and localization capability of different lightweight visual models under extremely small-scale target scenarios, particularly in real greenhouse environments where pest body sizes are extremely small, initial lesion areas are limited, and target-to-background contrast is low. Through unified small-object sample partitioning and consistent inference settings, model stability and recall capability in fine-grained recognition tasks were systematically measured.

As shown in Table 3, Light-HortiNet demonstrated a superior trade-off between detection precision and deployment efficiency. It achieved the highest values in mAP-small and recall-small, while maintaining competitive inference speeds with a latency of 43.8 ms and a controlled memory footprint of 14.5 MB. In terms of energy efficiency, the model operated at approximately 6.2 W, striking a favorable balance suitable for battery-powered edge devices. YOLOv11n exhibited relatively superior performance among the YOLO family, primarily benefiting from its updated feature fusion strategy which enabled stronger contextual modeling; however, this came at the cost of slightly elevated latency and memory usage compared to our method. YOLOv8n maintained stable performance, although its feature activation capability for extremely small targets remained constrained. YOLOX-Tiny and MobileNetV3 were oriented toward extreme lightweight design, achieving the lowest power consumption and latency by reducing channel numbers and network depth, but this optimization strategy resulted in a noticeable decline in detection accuracy. MobileViT enhanced global modeling capability by introducing a lightweight transformer structure, but significant memory overhead and loss of local high-frequency texture preservation were observed. EfficientDet-D0 incorporated bidirectional feature fusion mechanisms, yet semantic transmission capability was limited under extremely small target scales. Tiny-DETR was constrained by the representational bottleneck of the set-matching detection mechanism in low-resolution embedding spaces, leading to the highest latency (56.8 ms) and memory usage (24.1 MB) among the evaluated models, resulting in weaker overall performance in both speed and accuracy.

**Figure 9:**
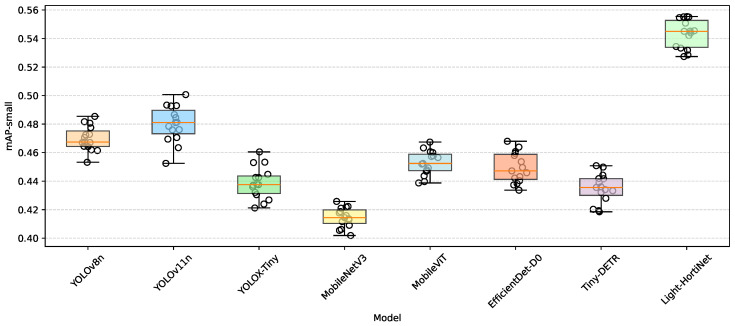
Small-object detection performance comparison.

From the perspective of mathematical characteristics, small-object detection tasks inherently require the preservation of more local high-frequency information in high-dimensional feature spaces and the avoidance of excessive smoothing of feature energy during multi-scale mapping, as shown in Figure 9. Traditional convolution-stacking-based models exhibit exponentially expanding receptive fields as network depth increases, which facilitates semantic modeling but progressively attenuates micro-structural response intensity during downsampling. Attention-based models under lightweight constraints tend to compress the attention computation space, resulting in sparse feature correlation matrices that are insufficient to establish stable global dependency relationships for micro-scale targets. The significant advantage of light-hortinet was attributed to the introduction of a dedicated small-object enhancement mechanism in the feature mapping process, enabling the formation of high-resolution feature preservation pathways at shallow stages, while cross-scale attention modulation suppressed the interference of background noise on discriminative boundaries. Consequently, steeper response gradients and clearer inter-class margins were formed in the feature distribution space. This structural advantage at the mathematical level enabled more compact feature clustering for small-object samples, resulting in superior recall and precision performance compared with conventional lightweight networks.

### 4.4. Cross-Domain Generalization Analysis

To thoroughly evaluate the robustness of the proposed model under varying environmental conditions, we conducted a domain-specific performance analysis. The test set consists of two distinct data sources: the In-situ Field Set, characterized by complex backgrounds, variable illumination, and occlusion typical of real greenhouse production; and the Public Source Set, which generally contains cleaner backgrounds and simpler compositions. Quantifying the performance gap between these two domains is critical for assessing real-world deployment feasibility.

Table 4 presents the detection performance across the two domains. It is observed that all models achieve higher accuracy on the Public Source Set, reflecting the lower difficulty of these samples. However, significant performance degradation occurs when models are applied to the In-situ Field Set. For instance, MobileViT and EfficientDet-D0 experience a sharp decline in mAP@50 (Domain Gap of −9.7% and −9.8%, respectively), indicating their limited ability to handle environmental noise such as leaf occlusion and water vapor interference. In contrast, Light-HortiNet demonstrates superior generalization capability. It not only achieves the highest absolute performance on the challenging field data (mAP@50=0.854) but also maintains the smallest performance gap (Δ=−4.1%) between domains. This robustness can be attributed to the synergistic effect of the CSLA module, which effectively filters background redundancy, and the specific data augmentation strategies (e.g., Mixup and Copy-paste) that simulate complex occlusions during training, thereby forcing the model to learn invariant pest features rather than relying on simple background correlations.

### 4.5. Ablation Studies

#### 4.5.1. Impact of Proposed Network Modules

The objective of this ablation experiment was to systematically verify the independent contributions and synergistic gain effects of the three proposed core modules on overall network performance. By progressively introducing the CSLA, BLSD, and SOEB modules, the variation trends of detection accuracy, small-object recognition capability, and inference efficiency under different combination strategies were compared, thereby revealing the actual impact of each module on the model representation capability and computational overhead.

As shown in Table 5, when only the backbone network was employed, values of 0.804 and 0.421 were achieved for mAP@50 and mAP-small, respectively, indicating that basic target recognition capability was obtained, while perception of small-scale targets remained limited. After the CSLA module was introduced, the overall detection accuracy increased to 0.839, and small-object performance was also noticeably improved, demonstrating that the cross-scale attention mechanism effectively enhanced information interaction among multi-scale features. With the introduction of the BLSD module, further improvements were observed in both mAP@50 and small-object metrics, reflecting the positive role of block-level distillation in enhancing semantic abstraction capability. When the SOEB module was introduced independently, the most significant improvement was observed in small-object performance, and the increase in mAP-small exceeded that of the overall mAP, confirming that the high-resolution small-object enhancement branch exerted a stronger amplification effect on micro-structural information. Pairwise combinations of different modules consistently exhibited stable performance superposition characteristics, while the simultaneous activation of all three modules achieved the optimal result, with mAP@50 increasing to 0.872 and mAP-small reaching 0.536, while acceptable inference frame rates were maintained, thereby validating the synergistic design of the overall architecture.

#### 4.5.2. Impact of Data Augmentation Strategies

To further evaluate the necessity of the proposed data processing pipeline, a stepwise ablation study was conducted on the data augmentation strategies. The experiment started with the raw dataset and sequentially incorporated Basic Augmentation (color jitter, random occlusion), Advanced Geometric Augmentation (Mosaic, Mixup), and the Small-object Copy-paste strategy. The complete Light-HortiNet architecture was used for all tests to isolate the contribution of data quality to the final performance.

As presented in Table 6, the model trained on raw data achieved a relatively low mAP-small of 0.384, indicating severe overfitting to simple background patterns and insufficient learning of pest features. The introduction of Basic Augmentation improved mAP@50 by 3.9%, confirming that color jitter and random occlusion effectively simulated the complex illumination and leaf occlusion variance typical of greenhouse environments, thereby forcing the network to learn more robust features. Subsequently, the inclusion of Mosaic and Mixup strategies resulted in a significant performance leap, with mAP@50 rising to 0.836. This suggests that enriching scene combinations and smoothing label distributions greatly enhanced the model’s spatial generalization capability. Finally, the application of the Small-object Copy-paste strategy yielded the most critical gain for fine-grained detection, boosting mAP-small from 0.468 to 0.536. This substantial increase demonstrates that increasing the occurrence frequency and spatial distribution of micro-scale targets effectively alleviates the class imbalance problem, ensuring that the lightweight model maintains high sensitivity to tiny pests like whiteflies and thrips even in complex scenes.

#### 4.5.3. Parameter Sensitivity Analysis

To determine the optimal hyperparameter settings for the proposed architecture, we conducted sensitivity analyses on two critical configuration sets: the low-rank compression ratio in the CSLA module and the multi-scale kernel combinations in the SOEB module.

**Impact of Low-rank Compression Ratio.** The rank parameter *r* in the Cross-Scale Lightweight Attention (CSLA) module controls the dimensionality of the feature subspace approximation (defined as C/r). A smaller compression ratio (larger *r*) preserves more semantic information but increases computational cost, while a larger ratio reduces redundancy but may lead to feature collapse. The theoretical suitability of this low-rank constraint for greenhouse scenarios stems from the intrinsic sparsity of pest features relative to the complex background. In typical greenhouse imagery, visual information is dominated by repetitive background patterns (e.g., leaf textures and soil), while salient targets (pests and lesions) occupy a low-dimensional manifold within the feature space. By enforcing a low-rank approximation, the CSLA module effectively acts as a semantic filter that suppresses high-frequency background noise and forces the attention mechanism to focus on the principal structural components of the targets, thereby enhancing feature purity without requiring dense full-rank computation. As shown in Table 7, we evaluated reduction ratios of 2,4,8, and 16. The results indicate that a ratio of 4 achieves the best trade-off, yielding a high mAP@50 of 0.872 with a moderate FLOPs increase. Although a ratio of 2 slightly improves accuracy (0.874), it disproportionately increases the model size and FLOPs, reducing FPS to 20.1. Conversely, excessive compression (ratio 16) causes a significant drop in accuracy (0.855), failing to capture sufficient fine-grained details.

**Impact of Multi-scale Kernel Combinations.** The Small-object Enhancement Branch (SOEB) relies on depthwise convolutions with varying kernel sizes to capture features at different receptive fields. We compared the default combination {3×3,5×5,7×7} against single-scale and other multi-scale variants. As presented in Table 8, using a single 3×3 kernel limits the receptive field, resulting in poor detection of larger pests or clustered targets (mAP@50: 0.851). The combination of {3,5,7} provides the most robust performance (mAP@50: 0.872), effectively covering the 5–20 pixel scale range of typical greenhouse pests. Interestingly, increasing kernel sizes further to {5,7,9} did not yield significant accuracy gains (0.873) but noticeably increased latency, validating that the {3,5,7} combination is the most efficient design for this specific task.

### 4.6. Discussion

In multi-platform deployment experiments, Light-HortiNet exhibited stable execution efficiency and favorable energy-performance characteristics across heterogeneous embedded computing environments. On the Raspberry Pi 4B platform (ARM Cortex-A72, 4 GB RAM), after INT8 quantization and acceleration using the ONNX Runtime inference engine, the model achieved a stable throughput of 7.8–9.4 FPS at an input resolution of 640×640, with an average per-frame latency of approximately 106–128 ms. Under a typical operating power consumption of 2.9–3.4 W, the power-normalized throughput reached approximately 2.3–3.1 FPS/W, satisfying the continuous operation requirements of low-power static monitoring nodes in greenhouse environments.

On the Jetson Nano platform (128-core Maxwell GPU, 4 GB RAM), after TensorRT INT8 acceleration, the throughput increased to 18.6–22.3 FPS, with per-frame latency reduced to approximately 45–54 ms. The measured power consumption was maintained at 5.8–6.6 W, yielding an energy efficiency of 3.1–3.8 FPS/W, which was well suited for deployment in greenhouse mobile inspection robots and rail-based automatic inspection systems. On the Jetson Xavier NX platform (384-core Volta GPU), the throughput was further elevated to 31.2–36.7 FPS, and the latency was reduced to 27–32 ms, while achieving an energy efficiency of 2.8–3.4 FPS/W within a power envelope of 9–12 W. Overall, the experimental results demonstrated that the proposed method supported sustained low-power operation on ARM-based platforms and provided reliable real-time video stream processing capabilities on embedded GPU platforms, confirming its practical engineering value for resource-constrained agricultural Internet-of-Things applications.

To provide a comprehensive assessment of practical application costs, it is necessary to discuss implementation complexity, training stability, and potential failure modes. Regarding implementation complexity, while the inference architecture is streamlined, the training pipeline introduces significant engineering overhead due to the Block-level Substitution Distillation (BLSD) framework. This requires maintaining a synchronous teacher–student computational graph and precise feature alignment logic, which increases GPU memory usage during training compared to standard single-model training. In terms of training stability, the proposed dynamic substitution mechanism mitigates gradient vanishing, yet the model exhibits sensitivity to the decay schedule of the substitution probability. An overly aggressive decay rate can lead to feature collapse before the student network fully establishes its representational capacity, necessitating careful hyperparameter tuning. Finally, distinct failure modes were observed during stress testing. The lightweight backbone, constrained by its limited channel capacity, tends to produce merged bounding boxes in scenarios with high-density pest occlusion, resulting in undercounting. Additionally, the model shows reduced robustness against significant spectral shifts, such as those caused by specific narrow-band LED supplemental lighting, indicating a reliance on color consistency within the training domain.

### 4.7. Limitations and Future Work

Although the proposed Light-HortiNet has achieved satisfactory performance in greenhouse fruit and vegetable pest and disease recognition tasks, certain limitations remain at the practical application level. First, the model has been primarily trained and inferred based on two-dimensional visible-light images, and for some diseases with highly concealed or extremely weak early symptoms, the visual features in the visible spectrum remain insufficiently informative. Under extreme environmental conditions such as strong specular reflection, heavy fog, or severe occlusion, detection stability still has room for improvement. Second, we applied the model to continuous video sequences captured in a greenhouse environment. The model maintained high accuracy on stable frames, but exhibited instability under conditions such as rapid insect movement or violent leaf swaying caused by ventilation, exhibiting phenomena like bounding box jitter and intermittent missed detections (flickering). This indicates that frame-by-frame independent inference lacks the necessary temporal consistency required for dynamic monitoring. In addition, although the model has been optimized for edge devices, trade-offs between memory bandwidth and real-time performance may still arise on embedded platforms with lower power budgets. Future work will focus on multimodal information fusion and cross-scenario generalization. On the one hand, infrared, thermal, and hyperspectral sensing data will be introduced and jointly modeled with visible-light images to enhance robustness against concealed diseases and environmental interference. On the other hand, addressing the instability observed in our preliminary video tests, temporal modeling and video-level detection frameworks will be explored. Specifically, spatiotemporal evolution information of targets will be incorporated into a unified modeling system to mitigate detection jitter and strengthen dynamic perception capabilities for pest spreading trends and disease lesion expansion processes.

## 5. Conclusions

This study was oriented toward the challenges of early recognition of fruit and vegetable diseases and pests and real-time deployment under resource-constrained greenhouse environments, and a lightweight and high-precision light-hortinet recognition framework was constructed, providing a technically feasible pathway for intelligent and fine-grained greenhouse management. By introducing a cross-scale lightweight attention mechanism, a block-level substitution distillation strategy, and a small-object enhancement branch, key bottlenecks in conventional lightweight models, including insufficient perception capability for small-scale targets and constrained feature representation capacity, were effectively addressed. In comprehensive experiments, superior overall performance was achieved in disease and pest detection tasks compared with mainstream lightweight models, with mAP@50 reaching 0.872, mAP@50:95 reaching 0.561, classification accuracy reaching 0.894, and f1-score reaching 0.882, while in small-object detection tasks, mAP-small of 0.536 and recall-small of 0.589 were obtained. In addition, stable real-time inference capability was maintained on edge device platforms, enabling an effective balance between accuracy and efficiency. The experimental findings demonstrate that the proposed method exhibits innovation at the algorithmic level and significant promotion value at the practical application level, and reliable technical support can be provided for precise greenhouse disease and pest early warning, intelligent pesticide regulation, and digital production management, thereby establishing an important foundation for the intelligent upgrading of facility agriculture.

## Figures and Tables

**Table 1:** Dataset composition and data source statistics.

Crop Type	Disease/Pest Type	Image Source	Number of Images
Tomato	Downy mildew, early blight, powdery mildew	Greenhouse field	4200
Cucumber	Leaf spot, powdery mildew	Greenhouse field & Public datasets	3800
Strawberry	Gray mold, leaf blight	Greenhouse field & Public datasets	3100
Pepper	Anthracnose, leaf spot	Greenhouse field & Public datasets	2600
Mixed crops	Whitefly, aphid, thrips, spider mite	Greenhouse field & Public datasets	2900
Mixed crops	Mixed disease samples	Public datasets	2700
Mixed crops	Rare pest categories	Public datasets	1400
Total	–	–	20,700

**Table 2:** Performance comparison with lightweight baseline models. Data are presented as mean ± standard deviation. The symbol ‘*’ indicates a statistically significant difference (p<0.05) compared to the best-performing baseline. **Bold** represents the best result.

Model	mAP@50	mAP@50:95	Accuracy	F1-Score	FPS
YOLOv8n	0.823±0.012	0.512±0.015	0.845±0.011	0.831±0.013	21.5±1.2
YOLOv11n	0.835±0.010	0.528±0.014	0.853±0.009	0.842±0.011	20.9±1.1
YOLOX-Tiny	0.798±0.015	0.487±0.018	0.821±0.014	0.809±0.016	23.4±1.5
MobileNetV3	0.764±0.018	0.462±0.020	0.801±0.016	0.792±0.019	24.1±1.4
MobileViT	0.816±0.013	0.505±0.016	0.836±0.012	0.825±0.014	19.7±0.9
EfficientDet-D0	0.809±0.014	0.499±0.017	0.828±0.013	0.817±0.015	18.9±0.8
Tiny-DETR	0.788±0.016	0.476±0.019	0.812±0.015	0.801±0.017	17.6±1.0
Light-HortiNet (Ours)	0.872±0.008 *	0.561±0.010 *	0.894±0.007 *	0.882±0.009 *	22.8±1.3

**Table 3:** Small-object detection performance comparison with efficiency metrics. Data are presented as mean ± standard deviation. The symbol ‘*’ indicates a statistically significant difference (p<0.05) compared to the best-performing baseline. **Bold** represents the best result.

Model	mAP-Small	Recall-Small	FPS	FLOPs (G)	Latency (ms)	Power (W)	Memory (MB)
YOLOv8n	0.463±0.012	0.512±0.015	21.5±1.2	6.8	46.5±2.1	6.8±0.3	16.4
YOLOv11n	0.478±0.011	0.527±0.014	20.9±1.1	7.1	47.8±2.3	7.0±0.4	17.2
YOLOX-Tiny	0.441±0.014	0.495±0.016	23.4±1.5	5.9	42.7±1.9	6.1±0.3	13.8
MobileNetV3	0.418±0.016	0.472±0.018	24.1±1.4	5.2	41.5±1.8	5.5±0.2	11.2
MobileViT	0.456±0.013	0.508±0.015	19.7±0.9	6.4	50.8±2.5	6.5±0.3	21.5
EfficientDet-D0	0.449±0.015	0.501±0.017	18.9±0.8	6.9	52.9±2.6	6.9±0.4	18.3
Tiny-DETR	0.432±0.017	0.486±0.019	17.6±1.0	7.3	56.8±2.8	7.5±0.5	24.1
Light-HortiNet (Ours)	0.536±0.009 *	0.589±0.011 *	22.8±1.3	5.6	43.8±2.0	6.2±0.3	14.5

**Table 4:** Performance comparison on In-situ Field Data versus Public Source Data. Data are presented as mean ± standard deviation. The symbol ‘*’ indicates a statistically significant difference (p<0.05) compared to the baseline models. The “Domain Gap” (Δ) denotes the relative performance drop when shifting from the cleaner public domain to the complex field domain. **Bold** represents the best result.

Model	In Situ Field Set		Public Source Set		Domain Gap (Δ)
	mAP@50	mAP-Small	mAP@50	mAP-Small	
YOLOv8n	0.792±0.014	0.415±0.017	0.854±0.011	0.512±0.013	−7.3%
YOLOv11n	0.805±0.013	0.432±0.015	0.868±0.010	0.529±0.012	−7.2%
MobileViT	0.775±0.016	0.408±0.018	0.858±0.012	0.504±0.014	−9.7%
EfficientDet-D0	0.768±0.015	0.395±0.019	0.851±0.013	0.498±0.015	−9.8%
Light-HortiNet (Ours)	0.854±0.009 *	0.518±0.012 *	0.891±0.008 *	0.554±0.010 *	−4.1%

**Table 5:** Ablation study of different modules in Light-HortiNet. Data are presented as mean ± standard deviation. The symbol ‘*’ indicates a statistically significant difference (p<0.05) compared to the baseline and other ablation variants. **Bold** represents the best result.

Configuration	mAP@50	mAP-Small	FPS
Baseline (Backbone only)	0.804±0.015	0.421±0.018	24.6±1.5
Baseline + CSLA	0.839±0.013	0.465±0.016	23.8±1.4
Baseline + BLSD	0.846±0.012	0.472±0.015	23.5±1.3
Baseline + SOEB	0.852±0.011	0.491±0.014	23.1±1.2
CSLA + BLSD	0.861±0.010	0.503±0.013	22.9±1.2
CSLA + SOEB	0.865±0.009	0.519±0.012	22.7±1.1
BLSD + SOEB	0.868±0.009	0.526±0.011	22.6±1.1
CSLA + BLSD + SOEB (Ours)	0.872±0.008 *	0.536±0.010 *	22.8±1.2

**Table 6:** Ablation study of data augmentation strategies. Data are presented as mean ± standard deviation. The symbol ‘*’ indicates a statistically significant improvement (p<0.05) compared to the raw data and partial augmentation strategies.

Augmentation Strategy	mAP@50	mAP-Small
None (Raw Data)	0.753±0.018	0.384±0.021
+Basic (Color + Occlusion)	0.792±0.015	0.415±0.019
+Mosaic & Mixup	0.836±0.012	0.468±0.016
+Copy-paste (Full Pipeline)	0.872±0.008 *	0.536±0.010 *

**Table 7:** Sensitivity analysis of the low-rank compression ratio in CSLA. Data are presented as mean ± standard deviation. The symbol ‘*’ indicates the selected optimal configuration based on the trade-off between accuracy and efficiency.

Compression Ratio (C/r)	mAP@50	mAP-Small	FLOPs (G)	FPS
2	0.874±0.007	0.539±0.009	6.8	20.1±1.0
4 (Default)	0.872±0.008 *	0.536±0.010 *	5.6	22.8±1.2
8	0.864±0.009	0.518±0.011	5.1	23.5±1.3
16	0.855±0.012	0.495±0.014	4.9	24.2±1.4

**Table 8:** Sensitivity analysis of depthwise convolution kernel combinations in SOEB. Data are presented as mean ± standard deviation. The symbol ‘*’ indicates statistically significant performance (p<0.05) compared to single-scale or smaller kernel combinations.

Kernel Combination	mAP@50	mAP-Small	Params (M)	FPS
{3}	0.851±0.014	0.488±0.016	2.8	24.5±1.5
{3,5}	0.863±0.011	0.512±0.013	3.0	23.6±1.4
{3,5,7} (Default)	0.872±0.008 *	0.536±0.010 *	3.2	22.8±1.2
{5,7,9}	0.873±0.007	0.538±0.009	3.5	21.2±1.1

## Data Availability

The original contributions presented in this study are included in the article. Further inquiries can be directed to the corresponding author.
